# Cases of Morbid Anatomy

**Published:** 1819-07-01

**Authors:** Edward Carbutt


### CASES of MORBID ANATOMY.

By Edward Carbutt, M.D.

(Continued from the Annals of Medicine and Surgery, Vol. II. p. 364.)

### Case 2.* *Bronchitis in a Child.*

NOVEMBER 8, 1817, Ann Wadsworth, aged 11 months, was this day admitted a Home-patient of the Manchester Infirmary and Dispensary. When I went to see her I found her living in a cellar, which was remarkably damp, or rather wet.

Her *Symptoms* were as follow:—She breathed very quick, with a slight hoarse sound upon each expiration; had an occasional cough, which was soft; *apparently* no expectoration; pulse was rapid and weak; countenance pale; tongue foul; stools green.

* Of Case 1. Organic Disease (Aneurism) of the Heart, the only one published in the Annals of Medicine and Surgery, a sufficient Abstract is to be found in the Medico-Chirurgical Journal, Vol. I. New Series, pages 118 and 119.

This was the 7th day of the complaint. What had been previously done, I could not learn, except that one or two leeches, had been applied to the thorax.

Imponatur pectori statim emplastrum lyttæ:—Ineat balneum tepidum bis quotidie.

℞. Hydrargyri submuriatis gr. j. Pulveris antimonialis gr. ß. Pulveris tragacanthæ compositi, gr. iij. Misce. Sumatur octavis horis.

9,10. Remains nearly as before, but apparently rather easier.

Pergat ut antea; admoveatur autem pectori cataplasma domesticum.

11,12. The disease continues with little variation. Pergat ut antea præscriptum est.

℞. Syrupi rhæados f ℥ii ß. Oxymellis scillæ f ʒiij. Misce. Sumatur cochleare minimum subinde, urgente tussi.

13,14. Continuentur medicamina.

15. She died early this morning. An *inspection* took place about thirty hours after death. There were present Mr. Jordan, Surgeon and Lecturer on Anatomy in this town, Mr. Boutflower, House-surgeon of the Infirmary, and myself.

In the right cavity of the thorax we found about half an ounce of serous fluid, without any flakes of coagulable lymph. The lungs had a very singular appearance, being principally of a cream-colour; but this colour was in small oval patches, even with the surface, and inferior to the serous membrane.* At the under surface of the right lung there appeared a deposition of blood similar to ecchymosis. On handling this lung, a crepitus was perceptible. Upon cutting into the lungs and slightly pressing, there exuded a whitish opake fluid, which was evidently not pus, but rather puriform mucus; and, upon dividing the trachea, a similar fluid was found in it in considerable quantity, but without any other marks whatever of inflammation in the internal membrane. In the pericardium there were two or three drachms of serum. The abdominal viscera we were not allowed to inspect. E. C.

---

* Dr. Carbutt was kind enough to transmit, with this case, a very beautiful drawing of a portion of one of the lungs, sliced off with a scalpel, and delineated with great graphic skill, by Mr. W. Brigham, surgeon, Manchester, which we regret that we cannot give a plate of, without risk of offending several other friends who have transmitted graphic representations of morbid parts about the same time. Edit.

